# Optimizing Liver Transplant Allocation for Hepatocellular Carcinoma: Development and Validation of a Survival Benefit‐Based Model

**DOI:** 10.1111/ctr.70488

**Published:** 2026-02-25

**Authors:** Hao Liu, Isabel Neckermann, Jason Mial‐Anthony, Charbel Elias, Abiha Abdullah, Vrishketan Sethi, Christopher Kaltenmeier, Amaan Rahman, Eishan Ashwat, Packiaraj Godwin, Subedi Sabin, Timothy Fokken, Shwe Han, Xingyu Zhang, Stalin Dharmayan, Jaideep Behari, Stela Celaj, Michele Molinari

**Affiliations:** ^1^ J.C. Walter Jr. Transplant Center and Department of Surgery Houston Methodist Hospital Houston Texas USA; ^2^ Department of Surgery University of Pittsburgh Medical Center Pittsburgh Pennsylvania USA; ^3^ Department of Surgery Georgetown University Washington D.C. USA; ^4^ School of Health and Rehabilitation Sciences University of Pittsburgh Pittsburgh Pennsylvania USA; ^5^ Department of Medicine University of Pittsburgh Pittsburgh Pennsylvania USA

**Keywords:** hepatocellular carcinoma, liver transplantation, model for end‐stage liver disease, organ allocation, predictive model, transplant‐associated survival benefit, tumor burden score, waitlist mortality

## Abstract

**Introduction:**

Liver transplantation (LT) is the only curative option for patients with unrespectable hepatocellular carcinoma (HCC). In the United States. current organ allocation policies grant the same priority to patients with tumors within the Milan criteria. This uniform approach leads to higher waitlist dropout among candidated with more advanced tumors of with more aggressive tumor biology. A model to stratify HCC candidates into different risk groups could optimize organ allocation by providing priority to patients within transplantable criteria but at increased risk of dropout.

**Methods:**

Data from 30,565 adult HCC LT candidates within the Scientific Registry of Transplant Recipients (SRTR) (2002–2022) were used. Inclusion criteria were age ≥18 years and tumors within Milan criteria. Recipients of previous transplants, multi‐visceral grafts, and those with missing exception applications for HCC were excluded. The population was randomly divided into development (*n* = 15,282) and validation (*n* = 15,283) cohorts. The primary outcome was 5‐year LT survival benefit, defined as the difference in survival with and without LT.

**Results:**

C‐MELD 3.0, serum AFP, and tumor burden score (TBS) were the strongest predictors of LT survival benefit. The HCC‐Liver Transplant Survival Benefit model was defined as HCC‐LTSB = 0.65 × (C‐MELD 145 3.0 − 6) + 1.99 × (TBS − 2.25) + 0.68 × log2(AFP). Validation demonstrated strong performance (Pearson's *r* = 0.93; 95% CI: 0.93–0.94; *R* ^2^ = 0.87; C‐index = 0.91).

**Conclusion:**

The HCC‐LTSB model accurately predicted the survival benefit provided by LT in candidates listed with unresectable HCC within UNOS criteria.

## 1 Introduction

Liver transplantation (LT) is the only curative treatment for patients with unresectable hepatocellular carcinoma (HCC) within specific limits such as the Milan Criteria or the University of San Francisco Criteria [1]. In the United States, current allocation policies assign the same priority to candidates with tumors that satisfy the United Network of Organ Sharing (UNOS) criteria [1]. Studies have consistently shown that larger or more aggressive tumors, despite still being within acceptable limits for transplantation, put patients at a significantly higher risk of waitlist dropout in comparison to candidates with smaller or more indolent disease [2, 3]. As a result, current policies fail to uphold the “Principle of Equity” by not adequately prioritizing patients at the highest risk of losing transplant eligibility. This phenomenon is in contrast with organ allocation systems in place for benign conditions, as patients at increased risk of death without LT receive priority to access deceased donor grafts.

Recently, Liu et al. [2] demonstrated that, among LT candidates in the United States, those at the highest risk of becoming ineligible because of tumor progression derive the greatest survival benefit from transplantation. These findings, also confirmed by other groups [2, 3, 4, 5], suggest that incorporating measures of oncologic urgency into allocation policy could both reduce waitlist dropout and maximize the survival benefit achieved through LT.

Over the past decade, growing evidence has highlighted the need to change organ allocation policies for HCC and provide different priority based on biological and oncological characteristics that differ among candidates [4, 6, 7, 8, 9, 10, 11]. In the United States, despite multiple proposed models, none has been implemented in clinical practice because of concerns over reliability, complexity and feasibility [4, 6, 7, 8, 9, 10, 11].

Nevertheless, reducing inequities in access to LT and maximizing the survival benefit derived from a limited supply of donor grafts remain central priorities for the transplant community. As with candidates transplanted for nonmalignant indications, urgency‐based prioritization should also be applied to patients with HCC to improve equity and clinical outcomes [12]. Ideally, such prioritization would rely on objective, reproducible, and widely available predictors integrated into a parsimonious and intuitive model capable of estimating both pre‐ and posttransplant survival. To date, however, no existing models fully meet these criteria.

To address this gap, we developed and internally validated the hepatocellular carcinoma Liver Transplant Survival Benefit (HCC‐LTSB) model, which quantifies the survival benefit of transplantation as the area between the survival curves of transplanted candidates and comparably listed candidates who do not undergo transplantation. By explicitly integrating both pre‐ and posttransplant outcomes, this model provides a biologically grounded estimate of transplant urgency and offers a more comprehensive framework for optimizing liver graft allocation among patients with unresectable HCC.

### 1.1 Patients and Methods

This retrospective study was conducted and reported following the TRIPOD (Transparent Reporting of a Multivariable Prediction Model for Individual Prognosis or Diagnosis) guidelines [13].

### 1.2 Consent and Reporting

The Institutional Review Board (IRB) of the University of Pittsburgh waived the requirement for informed consent (protocol number PRO: 13060220) because of the study's observational design and use of anonymized data. This study adhered to the ethical standards set forth in the 2013 revision of the Declaration of Helsinki and the Declaration of Istanbul on Organ Trafficking and Transplant Tourism [14], ensuring compliance with international research ethics.

### 1.3 Patient Population

Patients aged ≥18 years who underwent LT for HCC in the United States between March 2002 and December 2022 were eligible for inclusion. The study population was restricted to candidates who applied for HCC MELD exception points. Exclusion criteria included redo LT, multi‐visceral transplants, prior solid organ transplantation, and lack of an HCC exception application. Recipients of living donor liver transplantation (LDLT) were excluded because many do not meet UNOS criteria for MELD exception points and are not subject to deceased donor allocation policies, and because their access to transplantation is often facilitated by the availability of a dedicated donor, resulting in minimal waitlist exposure. The final cohort was randomly divided into development and validation samples of approximately equal size using random assignments to ensure a robust model evaluation.

### 1.4 Data Source

Demographic and clinical data were obtained from the Scientific Registry of Transplant Recipients (SRTR), a comprehensive national database that collects detailed information on organ donors, waitlisted candidates, and transplant recipients in the United States. These data are submitted by members of the Organ Procurement and Transplant Network (OPTN). The Health Resources and Services Administration (HRSA), an agency within the U.S. Department of Health and Human Services, oversees the activities of both OPTN and SRTR contractors, ensuring the integrity, accuracy, and reliability of the data.

### 1.5 Data Collection

The following variables were extracted from the SRTR: recipient demographics (age, sex, race, ethnicity, and blood group), clinical characteristics (presence of diabetes, dialysis history, tumor size and number, serum alpha fetoprotein (AFP) levels, and calculated C‐MELD score), and operative parameters (cold ischemia time). Follow‐up data included listing date, transplantation date, date of the last follow‐up, waitlist dropout, and death. The donor characteristics included age, sex, and ethnicity. Patients were followed up until death, the date of the last recorded follow‐up, or the study's end date, whichever occurred first. Missing data were handled using multiple imputation methods when appropriate. Variables with >10% missing data were excluded from analysis.

### 1.6 Outcomes and Predictors

Our primary aim was to develop a model for predicting the Liver Transplant Associated Survival Benefit (LTSB) in patients with unresectable HCC listed for LT. LTSB was defined as the difference in the area under the survival curves (AUC), restricted to five years, starting at the time of listing between patients who underwent LT and similar patients who did not receive LT [15]. Survival curves were generated using adjusted multivariable Cox regression models, and LTSB was measured in months.

Waitlist dropout was defined as removal from the waitlist due to worsening health, tumor progression beyond the transplant criteria, or death. Patients who dropped out were followed until death using data available from the Social Security Administration (SSA) dataset and OPTN survival data reported in the SRTR registry or the study's termination date, whichever occurred first.

The Tumor Burden Score (TBS) was calculated using the diameter of the largest HCC and the total number of HCCs identified in cross sectional radiological studies (computed tomography or magnetic resonance imaging) at the time candidates filed applications for MELD exception points [16, 17]. The formula for TBS, developed by Sasaki et al. [18], is:


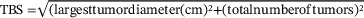

C‐MELD was derived using Kamath's equation [19] excluding exception points for HCC: C‐MELD = 3.78 × ln(serum bilirubin in mg/dL) + 11.2 × ln(INR) + 9.57 × ln(serum creatinine in mg/dL) + 6.43.

### 1.7 Statistical Analysis

Continuous variables were summarized as means with standard deviations (SD) or medians with interquartile ranges (IQR) for non‐normal distributions, while categorical variables were expressed as frequencies (percentages). Statistical comparisons were performed using the Student's t‐test, ANOVA, or Kruskal–Wallis test for continuous variables and *χ* ^2^ test or Fisher's exact test as appropriate.

Patients were followed up from listing until LT, waitlist dropout due to tumor progression, death, or delisting for other reasons. Overall survival was modeled using the Fine‐Gray competing risks approach, accounting for non‐transplant‐related mortality. The association between candidate characteristics and LTSB was evaluated using multivariable regression, with predictor selection informed by LASSO analysis and the Akaike Information Criterion (AIC) [20]. The Fine‐Gray competing risks approach was chosen over the traditional Cox regression because of its ability to account for non‐transplant‐related mortality as a competing risk, providing a more accurate estimation of the LT benefit. Candidate‐level covariates included age, sex, race/ethnicity, C‐MELD score, cause of liver disease, body mass index (BMI), diabetes history, dialysis status, TBS, serum AFP levels, and transplant status. C‐MELD was included as a primary predictor because of its established role in assessing liver disease severity and the risk of pre‐LT mortality [12]. AFP [8] and TBS [2] were selected based on their strong correlation with tumor biology and survival outcomes, as confirmed in previous studies.

### 1.8 LTSB and Development of the HCC‐LTSB Model

The LTSB was defined as the difference in the AUC between patients who underwent LT and those who did not, restricted to five years from listing, [2, 21]. For each transplanted patient, LTSB represented the difference between predicted 5‐year posttransplant survival and predicted 5‐year waitlist survival.

A mixed‐effects proportional hazards model was used to estimate survival functions, incorporating both recipient‐ and donor‐level covariates, with TBS included as a cluster‐specific random effect. Recipient variables included age, calculated C‐MELD score, sex, blood type, BMI, race/ethnicity, HCC risk factors, diabetes, dialysis status, and serum AFP levels at listing. Donor variables included age, sex, race/ethnicity, and cold ischemic time. The HCC‐LTSB score was constructed using multivariable regression, with coefficients reflecting each predictor's contribution to the estimated survival benefit. Associations between individual predictors and LTSB were assessed using Pearson correlation coefficients and scatter plots.

### 1.9 Validation and Sensitivity Analyses

Model performance was internally validated using cross‐validation, with discrimination assessed using shrunken *R* ^2^ coefficients to account for model complexity. *R* ^2^ values were classified as very high (0.82–1.00), high (0.49–0.81), moderately low (0.17–0.48), very low (0.05–0.16), or negligible (0–0.04).

To minimize bias and enhance policy applicability, age, sex, race/ethnicity, and primary HCC risk factors were omitted from the final HCC‐LTSB score. Sensitivity analyses confirmed that their exclusion did not meaningfully affect model performance.

Because HCC MELD exception point assignment has varied over time according to donation service area‐level median C‐MELD at transplant (MMaT), additional sensitivity analyses were performed across allocation eras. Model discrimination and calibration were evaluated before and after implementation of the National Liver Review Board and MMaT‐based exception scoring in May 2019 to assess robustness to temporal and regional variation in allocation practices.

### 1.10 Software and Statistical Significance

Statistical significance was defined as a two‐tailed *P* value <0.05, with Bonferroni correction applied for multiple comparisons when appropriate. All analyses were performed using R (version 3.6.3; R Core Team 2020) and IBM SPSS Statistics for Windows (version 28.0; Armonk, NY, USA).

## 2 Results

### 2.1 Study Population

**FIGURE 1:**
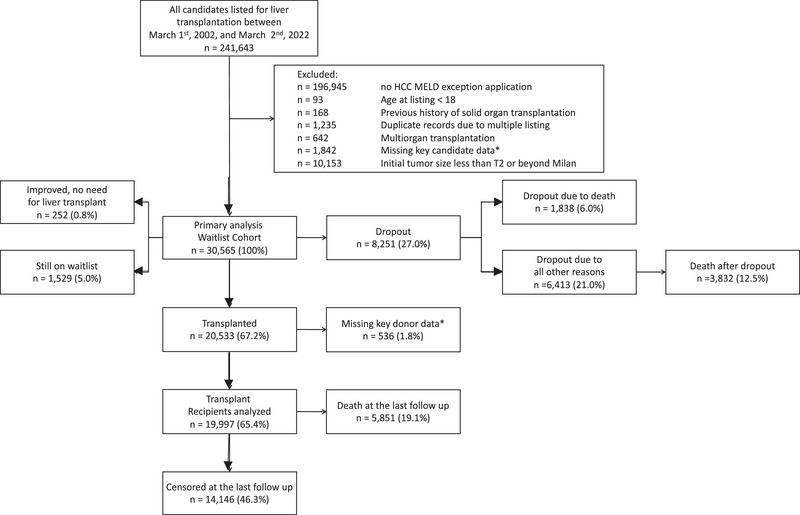
The flowchart illustrating the selection of the study population among all adult patients listed for LT with HCC in the United States between 2002 and 2022.

1, 2002, and March 2, 2022, a total of 241 643 adults were listed for LT in the U.S. Among them, 30 565 patients with HCC meeting the UNOS criteria for MELD exception points were eligible for inclusion. A detailed flowchart of the study population is shown in Figure 1. The demographic and clinical characteristics of the patients are summarized in Table 1. The study cohort was randomly divided into two equivalent groups: 15 282 candidates were allocated to the training cohort for HCC‐LTSB score development, whereas 15 283 patients comprised the validation cohort.

**TABLE 1:** Characteristics of the study population, stratified by waitlist outcomes.

Characteristics	Overall	Transplanted	Dropout
	*n*. 30,565	*n*. 19,997	*n*. 8,249
Age, years, mean (SD)	59.21 (7.47)	58.72 (7.47)	60.00 (7.06)
Sex = Male, *n* (%)	23400 (76.6)	15414 (77.1)	6304 (76.4)
BMI, mean (SD)	29.06 (5.43)	29.08 (5.31)	28.94 (5.60)
Recipient Race by SRTR, *n* (%)			
Asian	2529 (8.3)	1553 (7.8)	710 (8.6)
Black / African American	2698 (8.8)	1752 (8.8)	767 (9.3)
Hispanic	5249 (17.2)	3141 (15.7)	1616 (19.6)
Multiracial / Other	443 (1.4)	272 (1.4)	128 (1.6)
White / Caucasian	19646 (64.3)	13279 (66.4)	5028 (61.0)
Recipient blood group (%)			
A	11284 (36.9)	7430 (37.2)	2974 (36.1)
AB	1138 (3.7)	875 (4.4)	185 (2.2)
B	3916 (12.8)	2691 (13.5)	948 (11.5)
O	14227 (46.5)	9001 (45.0)	4142 (50.2)
History of Diabetes, *n* (%)	9923 (32.5)	6419 (32.1)	2701 (32.7)
Need for dialysis, *n* (%)	166 (0.5)	18 (0.1)	133 (1.6)
Viral hepatitis C (HCV), *n* (%)	16370 (53.6)	10989 (55.0)	4482 (54.3)
Viral hepatitis B (HBV), *n* (%)	1928 (6.3)	1276 (6.4)	485 (5.9)
Alcohol induced liver disease, *n* (%)	5919 (19.4)	3753 (18.8)	1686 (20.4)
Metabolic associated liver disease (MAFLD), *n* (%)	3280 (10.7)	2055 (10.3)	829 (10.0)
C‐MELD, median, (IQR)	10.00 (8.00, 14.00)	10.00 (8.00, 13.00)	11.00 (8.00, 14.00)
TBS, mean (SD)			
≤3	15371 (50.3)	10242 (51.2)	3857 (46.8)
>3 and ≤5	14575 (47.7)	9390 (47.0)	4184 (50.7)
>5	619 (2.0)	365 (1.8)	208 (2.5)
AFP, median, (IQR)	9.00 [4.00, 34.00]	9.00 [5.00, 30.00]	11.00 [5.00, 58.00]
AFP category, *n* (%)			
≤500	29279 (95.8)	19319 (96.6)	7700 (93.3)
501–1000	593 (1.9)	329 (1.6)	228 (2.8)
>1000	693 (2.3)	349 (1.7)	321 (3.9)
Time to drop out, median (IQR)	—	—	8.94 [4.53, 17.15]
Time to liver transplant, median (IQR)	—	6.90 [2.69, 12.16]	—
Donor age, years, mean (SD)	—	43.06 (16.64)	—
Donor sex = Male, *n* (%)	—	11987 (59.9)	—
Donor race by SRTR, *n* (%)			
Asian	—	617 (3.1)	—
Black / African American	—	3464 (17.3)	—
Hispanic	—	2689 (13.4)	—
Multiracial / Other	—	211 (1.1)	—
White / Caucasian	—	13016 (65.1)	—
Cold ischemic time, hours, mean (SD)	—	6.40 (2.83)	—

*Note:* SD = Standard deviation, C‐MELD = calculated model for end stage liver disease, AFP = alpha feto‐protein.

### 2.2 Development of the HCC‐LTSB Score

The HCC‐LTSB score was developed using a competing risk survival analysis based on the Fine‐Gray subdistribution hazard model to identify key predictors (Table 2). The dependent variable was LTSB, and competing events included death on the waitlist, death posttransplant, LT, and removal from the waitlist for reasons other than death.

**TABLE 2:** Fully adjusted Cox regression analysis, including interaction terms, for the risk of death after listing for LT in patients with unresectable HCC.

Characteristic	Coefficient	Hazard ratio	se(coef)	*Z*	*P*
Transplantation	−0.577	0.561	0.174	−3.323	<0.001
C‐MELD	0.084	1.087	0.005	18.613	<0.001
TBS	0.286	1.332	0.025	11.406	<0.001
log_2_AFP	0.118	1.125	0.007	17.131	<0.001
BMI	−0.009	0.991	0.004	−2.451	0.014
Blood type A					ref
Blood type AB	−0.002	0.998	0.133	−0.016	0.987
Blood type B	−0.051	0.950	0.066	−0.775	0.438
Blood type O	0.031	1.031	0.040	0.755	0.450
HCV	−0.024	0.977	0.043	−0.556	0.578
HBV	−0.461	0.631	0.094	−4.896	<0.001
Alcohol induced liver disease	−0.095	0.910	0.049	−1.938	0.053
MAFLD	−0.044	0.957	0.075	−0.585	0.558
Diabetes	0.151	1.163	0.042	3.634	<0.001
Dialysis	0.009	1.009	0.154	0.056	0.955
Interactions					
Transplantation: C‐MELD	−0.066	0.936	0.006	−10.218	< 0.001
Transplantation: TBS	−0.191	0.826	0.036	−5.278	< 0.001
Transplantation: log_2_AFP	−0.066	0.936	0.010	−6.376	< 0.001
Transplantation: BMI	0.006	1.006	0.005	1.196	0.232
Transplantation: Blood type A	−0.166	0.847	0.164	−1.008	0.314
Transplantation: Blood type AB	−0.034	0.966	0.089	−0.384	0.701
Transplantation: Blood type B	−0.014	0.986	0.057	−0.240	0.810
Transplantation: Blood type O	0.095	1.100	0.060	1.596	0.110
Transplantation: HCV	0.047	1.048	0.131	0.360	0.719
Transplantation: HBV	0.102	1.107	0.069	1.476	0.140
Transplantation: Alcohol use disorder	0.036	1.037	0.109	0.333	0.739
Transplantation: MAFLD	0.034	1.034	0.058	0.582	0.561
Transplantation: Diabetes	−0.338	0.713	1.015	−0.333	0.739

*Note:* se(coef) = standard error of the coefficient; AFP = alpha feto‐protein, BMI = body mass index, HCV = hepatitis C virus, HBV = hepatitis B Virus, C‐MELD = calculated model for end stage liver disease, TBS = tumor burden score, MAFLD = metabolic associated fatty liver disease.

**FIGURE 2:**
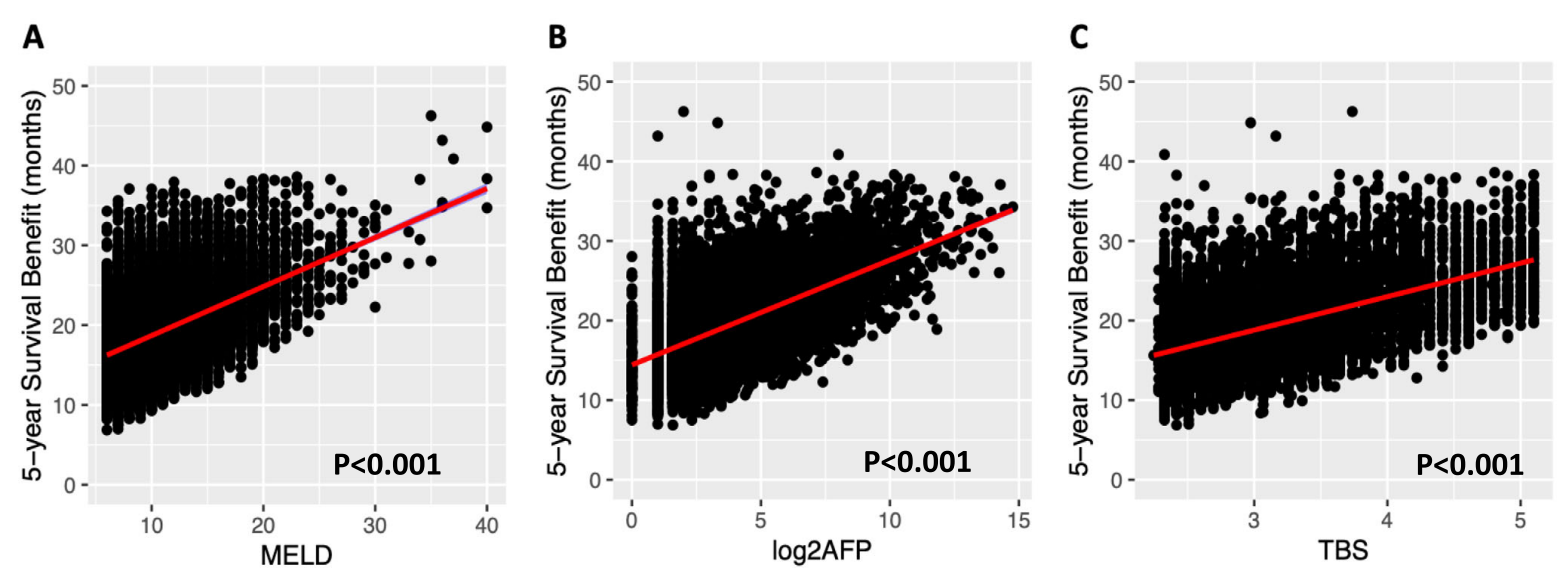
Scatter plots illustrating the associations between liver transplant—associated survival benefit (LTSB) and key predictors: calculated Model for End‐Stage Liver Disease (C‐MELD) score (Panel A), log_2_‐transformed alpha‐fetoprotein (log_2_AFP) (Panel B), and TBS (Panel C).

Feature selection using LASSO regression and AIC confirmed that the three strongest predictors were C‐MELD, TBS, and log‐transformed serum AFP (Log2AFP). Figure 2 illustrates Pearson's correlation coefficients between LTSB and these key variables.

- C‐MELD: *r* = 0.47 (95% CI 0.46–0.49; *R* ^2^ = 0.22; *P* < 0.001)
- Log2AFP: *r* = 0.57 (95% CI 0.55–0.58; *R* ^2^ = 0.32; *P* < 0.001)
- TBS: *r* = 0.55 (95% CI 0.54–0.57; *R* ^2^ = 0.31; *P* < 0.001)

The final HCC‐LTSB formula was developed using the regression coefficients of the Fine‐Gray model, and tested for nonlinear relationships using restricted cubic splines. The final formula is:

HCC‐LTSB = 0.65 × (C‐MELD − 6) + 1.99 × (TBS − 2.25) + 0.68 × Log_2_AFP.

### 2.3 Validation

**FIGURE 3:**
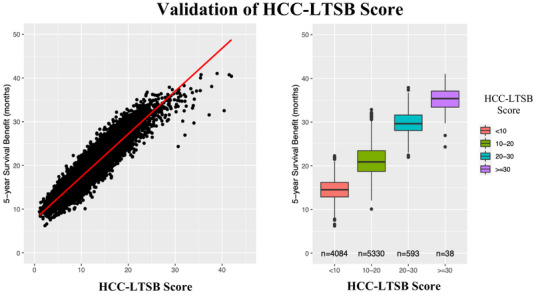
Scatter plot of the HCC‐LTSB score versus liver transplant—associated survival benefit (LTAB) in the validation cohort. Median LTAB (IQR) was 14.5 months (12.9–16.2) for HCC‐LTSB < 10, 20.9 months (18.7–23.4) for 10–<20, 29.6 months (28.1–31.6) for 20–<30, and 35.4 months (33.4–37.1) for ≥30.

The HCC‐LTSB score demonstrated strong predictive accuracy in the validation cohort, with a Pearson correlation coefficient of 0.93 (95% CI: 0.93–0.94; *R* ^2^ = 0.87; *P* < 0.001) (Figure 3A) and a concordance index (C‐index) of 0.91, indicating excellent discriminative ability. Calibration plots showed near‐perfect agreement between survival benefit estimates, with a significant dose‐response relationship across quartiles (*P* < 0.001 for all pairwise comparisons) (Figure 3B).

## 3 DISCUSSION

Current organ allocation policies in the United States apply uniform priority to all eligible candidates with HCC, largely independent of differences in tumor biology and oncologic risk factors [22]. While this approach was designed to standardize access to transplantation, it fails to account for heterogeneity in disease behavior, resulting in disproportionately higher waitlist dropout among candidates with more aggressive tumors [23]. As a result, patients with the greatest oncologic urgency may paradoxically derive the least benefit from the current allocation framework. These limitations have prompted increasing recognition of the need to move beyond static, criteria‐based policies toward biologically informed strategies that better reflect both urgency and expected benefit from LT.

In response to these challenges, several models, including HALT‐HCC, de‐MELD, C‐MELD‐Eq, OPOM, and more recently, multi‐HCC, have been proposed to refine prioritization among HCC candidates [4, 6, 8, 11, 22]. Although these models incorporate tumor‐related variables such as size, number, and serum AFP, their primary focus has been on predicting waitlist dropout rather than quantifying posttransplant outcomes. As a result, their ability to inform organ utilization and to prioritize candidates based on the survival benefit conferred by transplantation remains limited, particularly in the context of persistent organ scarcity [2, 11].

LTSB provides a distinct and complementary framework by quantifying the incremental survival gained from transplantation relative to remaining on the waitlist. LTSB is defined as the area between the survival curves of transplanted and non‐transplanted candidates, typically restricted to a five‐year horizon to facilitate standardized comparisons. To accurately estimate this metric in the presence of competing events such as waitlist dropout and non‐transplant‐related mortality, the Fine‐Gray subdistribution hazard model was employed. By directly modeling cumulative incidence rather than treating competing events as censoring, this approach offers a more realistic representation of clinical trajectories among LT candidates. The validity and superiority of this methodology have been consistently demonstrated in large registry‐based analyses, including studies derived from the SRTR, particularly in populations with HCC [5, 20, 23, 24, 25, 26, 27, 28, 29, 30, 31].

The HCC‐LTSB model was developed to operationalize this concept by integrating both pre‐ and posttransplant survival into a single, continuous metric. Unlike existing prioritization schemes that largely ignore tumor biology, HCC‐LTSB incorporates AFP, TBS, and C‐MELD—all well‐established, independent predictors of waitlist outcomes and posttransplant survival [4, 6, 8, 11, 22]. By combining these variables, the model estimates the net survival advantage attributable to transplantation. Accordingly, candidates with aggressive tumor biology and limited likelihood of surviving on the waitlist, yet favorable posttransplant prognosis, receive higher HCC‐LTSB scores, reflecting greater incremental benefit from timely LT. Conversely, candidates with indolent disease and relatively preserved waitlist survival derive a smaller marginal benefit and therefore receive lower scores.

Importantly, liver allocation in the United States remains fundamentally grounded in medical urgency, with organs prioritized for candidates at highest risk of death or removal without transplantation. The HCC‐LTSB model is not intended to replace this urgency‐based paradigm. Rather, it is designed to complement existing frameworks by providing an objective, biologically grounded estimate of transplant‐associated survival benefit. By aligning oncologic urgency with expected benefit, HCC‐LTSB offers a mechanism to inform refinement of HCC exception policies. Conceptually, this approach could be integrated with measures of waitlist dropout risk or incorporated as a weighting factor within emerging continuous distribution models, thereby preserving the primacy of urgency while improving equity and efficiency in the allocation of scarce liver grafts.

The relevance of HCC‐LTSB is further underscored by the ongoing transition toward continuous distribution in liver allocation. This policy evolution seeks to balance medical urgency, access, and efficiency through multidimensional scoring systems [32, 33]. Within this context, HCC‐LTSB has the potential to function as an oncologic analogue to MELD‐based urgency in nonmalignant liver disease, offering a transparent and reproducible method to capture biologic heterogeneity among HCC candidates and to inform prioritization decisions in a standardized manner.

The variable effectiveness of bridging therapies further highlights the limitations of static allocation approaches. Locoregional and systemic treatments are widely used to maintain transplant eligibility, yet responses vary substantially across patients [34, 35, 36, 37, 38]. Some individuals achieve durable disease control, whereas others experience progression despite therapy. Current allocation policies do not systematically differentiate between responders and non‐responders, potentially leading to misalignment between priority and true oncologic risk.

Although biologic variables such as age, sex, and race are known to influence transplant outcomes, they were intentionally excluded from the final HCC‐LTSB score to enhance policy feasibility and equitable implementation. Sensitivity analyses demonstrated that inclusion of these variables did not meaningfully improve discrimination or alter patient ranking by survival benefit. Their exclusion therefore reflects a deliberate design choice favoring objective, modifiable oncologic and biochemical predictors over non‐modifiable demographic characteristics, ensuring that the model remains suitable for national allocation policy rather than individualized prognostication.

This study has several important strengths, including its foundation on a large, nationally representative cohort of adult patients listed for LT for unresectable HCC, the absence of exclusions based on race, sex, or geographic region, and the use of advanced competing‐risk and survival benefit methodology. These features enhance generalizability and allow for robust estimation of transplant‐associated survival benefit in a population characterized by multiple competing clinical events. Nevertheless, limitations inherent to retrospective registry‐based analyses must be acknowledged, and residual confounding cannot be fully excluded.

Although comprehensive, the SRTR does not capture granular information on evolving systemic and locoregional therapies, longitudinal treatment response, or dynamic tumor biology. As a result, the impact of emerging oncologic strategies on transplant eligibility and posttransplant outcomes cannot be fully assessed. Future studies incorporating serial imaging, AFP kinetics, and molecular and histopathologic biomarkers may further refine biologic risk stratification and improve survival benefit estimation.

Allocation policy for HCC also evolved during the study period, particularly with the implementation of the National Liver Review Board and Median MELD at Transplant (MMaT)‐based exception point assignment in 2019, which introduced regional variability in access to LT. While these changes could theoretically influence transplant probability across eras, sensitivity analyses demonstrated no meaningful differences in discrimination or calibration of the HCC‐LTSB model before and after policy implementation. This temporal stability represents a key strength and supports the robustness of the model across allocation eras and geographic contexts.

Although the present study focused on quantifying transplant‐associated survival benefit, an important area for future investigation will be to evaluate whether the HCC‐LTSB score can also stratify waitlist dropout risk. Because urgency‐based allocation remains central to U.S. transplant policy, demonstrating an association between HCC‐LTSB and dropout would further support its relevance for exception policy refinement and integration into continuous distribution frameworks.

More broadly, HCC‐LTSB provides a flexible framework for future research, including assessment of allocation efficiency across regions and eras, evaluation of the incremental benefit of emerging therapies, and development of dynamic prioritization strategies incorporating longitudinal tumor response. Prospective validation and policy‐focused modeling studies will be essential to determine how survival benefit‐based instruments such as HCC‐LTSB can complement urgency‐based allocation and advance equity and efficiency in LT for unresectable HCC.

In conclusion, the HCC‐LTSB model provides a robust, clinically intuitive framework for estimating transplant‐associated survival benefits in patients with unresectable HCC. By integrating tumor biology with liver disease severity, it addresses key shortcomings of static allocation policies and offers a principled approach to aligning urgency with expected benefit. Prospective validation and thoughtful integration into continuous distribution frameworks will be essential to determine its ultimate clinical utility and policy relevance.

## Author Contributions

**Hao Liu**: literature search, study design, data collection, data analysis, data interpretation, and writing. **Isabel Neckermann**: literature search, study design, data collection, data analysis, and writing. **Jason Mial‐Anthony**: data interpretation, technical help, contribution as part of a large. **Charbel Elias**: data interpretation, technical help, contributing as part of a large. **Abiha Abdullah**: literature search, study design, data collection data analysis. **Vrishketan Sethi**: literature search, study design, data collection data analysis. **Christopher Kaltenmeier**: data interpretation, contributing as part of a large collaboration group. **Amaan Rahman**: data interpretation, technical help, manuscript writing. **Eishan Ashwat**: data interpretation, technical help, manuscript writing. **Packiaraj Godwin**: data interpretation, technical help, manuscript writing. **Subedi Sabin**: data interpretation, technical help, manuscript writing. **Timothy Fokken**: data interpretation, manuscript writing. **Shwe Han**: data interpretation, manuscript writing. **Xingyu Zhang**: study design, data collection, data analysis, statistical support. **Stalin Dharmayan**: data interpretation, writing, editing. **Jaideep Behari**: study design, data interpretation, manuscript writing, editing. **Stela Celaj**: study design, data interpretation, manuscript writing, editing. **Michele Molinari**: study design, statistical analysis, data interpretation, manuscript writing, and editing.

## Funding

The author has nothing to report.

## Conflicts of Interest

The authors declare no conflict of interest that could influence the objectivity, integrity, or interpretation of the research findings presented in this manuscript.

## Data Availability

The data that support the findings of this study are available on request from the corresponding author. The data are not publicly available due to privacy or ethical restrictions.
